# Integrating rumen microbiome and host metabolome to investigate feed conversion ratio across different fattening stages in Hu sheep

**DOI:** 10.5713/ab.260317

**Published:** 2026-06-01

**Authors:** Quanzhong Xu, Xiaoxue Zhang, Huibin Tian, Xiaobin Yang, Jian Zhang, Hongjian Li, Zongwu Ma, Deyin Zhang, Kai Huang, Yukun Zhang, Yuan Zhao, Xiaolong Li, Liming Zhao, Jiangbo Cheng, Dan Xu, Fadi Li, Xiuxiu Weng, Weiwei Wu, Weimin Wang

**Affiliations:** 1 State Key Laboratory of Herbage Improvement and Grassland Agro-Ecosystems, Key Laboratory of Grassland Livestock Industry Innovation, Ministry of Agriculture and Rural Affairs, Engineering Research Center of Grassland Industry, Ministry of Education, College of Pastoral Agriculture Science and Technology, Lanzhou University, Lanzhou, China; 2 College of Animal Science and Technology, Gansu Agricultural University, Lanzhou, China; 3 Key Laboratory of Genetics Breeding and Reproduction of Xinjiang Wool Sheep & Cashmere Goat, Institute of Animal Science, Xinjiang Uygur Autonomous Region Academy of Animal Science, Urumqi, China

**Keywords:** Fattening Stages, Feed Conversion Ratio, Host Metabolome, Hu Sheep, Rumen Metagenome

## Abstract

**Objective:**

Feed conversion ratio (FCR) is a crucial economic trait in animal breeding and management and is also important for environmental sustainability. This study aimed to investigate the putative regulatory mechanisms of FCR in sheep by integrating rumen microbiota and host metabolome through multi-omics analysis.

**Methods:**

FCR data were collected from 127 male Hu sheep. Extreme individuals were selected for rumen metagenomic and serum metabolomic analyses to identify key factors driving FCR across early and late fattening stages.

**Results:**

*Bacteroides*, *Prevotella*, and other genera were identified as dominant taxa in the rumen across both stages, suggesting their ipotential role in FCR regulation. Notably, *Nocardia tengcongensis* differed significantly between the highest FCR values (HF) and lowest FCR values (LF) groups at different stages, indicating its potential as a predictive biomarker of feed efficiency. Functional analysis revealed that the pentose phosphate pathway (M00004) and lysine biosynthesis via the succinyl-DAP pathway (M00016) were enriched in the LF group, whereas the methanogenesis pathway (M00357) was significantly enriched in the HF group, indicating increased methane production. Thirteen metabolites consistently differed between HF and LF across fattening stages and they may be used as predictive biomarkers. Furthermore, the abundance of *Prevotella* and *Bacteroides* increased over time and showed significant correlations with key metabolites.

**Conclusion:**

These findings suggest strong interactions between rumen microbiota and host metabolites that may collectively influence FCR, providing new insights into microbial and metabolic regulation of feed efficiency and a theoretical basis for optimizing feeding strategies in sheep.

## INTRODUCTION

With the growth of the global population and a dietary shift towards animal protein, the livestock industry is under significant pressure to improve production efficiency [1,2]. Feed costs account for approximately 70% of the total expenses in sheep production [3], making the optimization of the feed conversion ratio (FCR) crucial for enhancing profitability and environmental sustainability. FCR is a key measure of an animal’s efficiency in converting feed into body weight (BW) gain, defined as the amount of feed required per unit of BW increase. A higher FCR value indicates lower feed efficiency, whereas a lower FCR value reflects higher efficiency. Studies indicate that animals with high feed efficiency not only gain weight more rapidly with less feed but also experience shorter fattening periods, thus improving overall feeding efficiency [4,5]. Greater FCR efficiency also implies reduced feed intake, which lowers greenhouse gas emissions, particularly methane, thereby contributing to the development of low-carbon livestock farming. Enhancing FCR can not only reduce livestock production costs and improve economic outcomes, but also provide an effective response to the anticipated rise in global dietary demands, while mitigating environmental pressures [6,7]. Therefore, optimizing FCR in livestock production systems holds considerable practical importance, promoting sustainable industrial growth and offering viable solutions to global food and environmental challenges.

The rumen is a hallmark organ of ruminants, and is an anaerobic, methanogenic fermentation chamber containing a dense and diverse microbial community [8]. The rumen microbial community consists of bacteria, archaea, protozoa and fungi, and is characterized by high population density, complex interspecies interactions [9,10]. A growing body of research suggests that comprehensive sequencing of the microbiome of gut microorganisms and joint analysis of this data with the host’s genome, transcriptome, and metabolome reveals relevant regulatory mechanisms for the host’s life activities [11], and promote productive performance [12]. In addition, rumen microbes affect feed efficiency in dairy cows [13]. Studies have shown that biomarkers can be used as indicators for the prediction of relevant traits. Several biomarkers in the early lactation phase have been shown to predict yield throughout lactation [14]. Such studies particularly used blood BHB or NEFA concentrations to diagnose metabolic health [15–18]. However, studies on FCR prediction during the fattening period are still scarce. Accurate prediction of FCR in the early fattening stage is vital for controlling farming costs. It empowers farmers to optimize feeding strategies and enhance economic efficiency throughout the entire fattening period. Therefore, the study of rumen microorganisms and blood metabolites is important for studying FCR in sheep.

In this study, rumen fluid and blood samples were collected at both the early and late fattening stages for microbiome and metabolome analyses to investigate their relationship with FCR, with the goal of providing a scientific basis for future feeding management strategies.

## MATERIALS AND METHODS

### Experimental animals and samples collection

**Figure 1:**
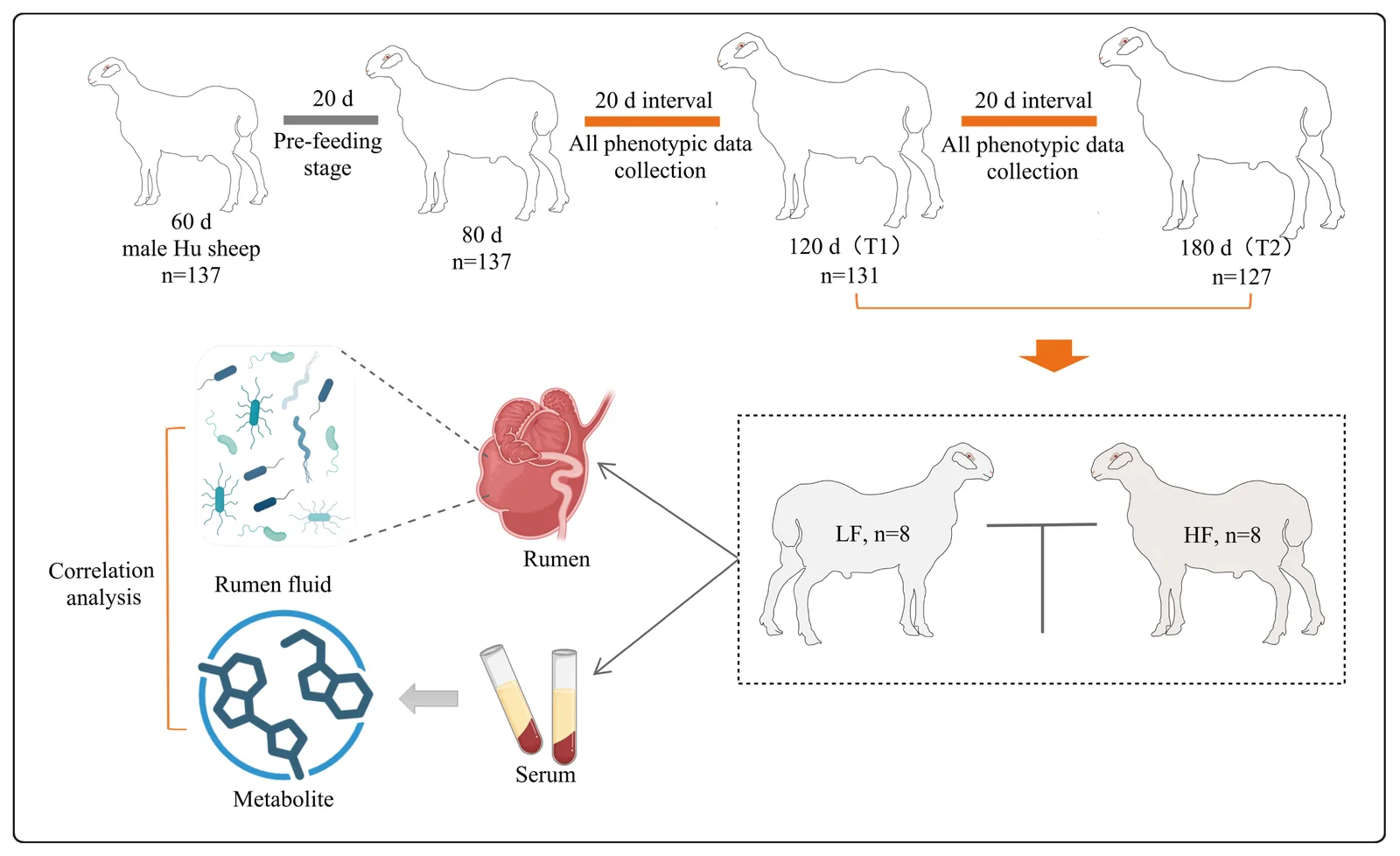
The experimental design depicting collection of samples and data analysis. LF, lowest FCR group; HF, highest FCR group; FCR, feed conversion ratio.

This study was conducted at a sheep farm in Minqin County, Wuwei City, Gansu Province, China, an experimental site of Lanzhou University. 137 Hu sheep rams were selected as candidate sheep. Candidate sheep were initially subjected to a standardized intensive feeding regimen. Subsequently, each animal was housed in an individual pen (0.8×1.0 m) within a well-ventilated, semi-open barn and maintained on a uniform diet throughout the experimental period (Supplement 1). Animals were fed ad libitum twice daily (08:00 and 18:00), with free access to clean water provided via an automatic watering system. BW and feed intake for each individual were recorded every 20 days using a calibrated livestock scale, between 06:00 and 08:00 a.m., prior to the morning feeding, from 80 to 180 days of age. During the experiment, we observed the health condition of all test sheep every day and eliminated the test sheep with poor physical condition over time (n = 10). The design of the study is presented in Figure 1. Eight animals with the highest and lowest FCR values were selected as HF and LF, respectively, for multi-omics analysis. Blood samples were collected via venipuncture using a blood collection needle, and rumen fluid was extracted with a negative pressure pump from all sheep at day 120 (T1) and day 180 (T2). These two time points were selected to represent distinct fattening stages in Hu sheep. Specifically, 120 days of age (T1) corresponds to the early fattening stage, characterized by rapid growth and increasing feed intake, whereas 180 days of age (T2) represents the late fattening stage, during which growth rate stabilizes and feed efficiency becomes a key determinant of production performance. One of the tubes of blood was analyzed by IDEXX ProCyte Dx Hematology Analyzer to determine the physiological parameters of blood. The other tube of blood sample were allowed to clot at room temperature for 30 minutes and then centrifuged at 3,000×g at 4°C for 10 min to separate the serum. The collected serum and rumen fluid were subsequently stored at −80°C for later analysis.

### DNA extraction, metagenome sequencing, and metagenomics data processing

Total microbial genomic DNA was extracted using the Mag-Bind Soil DNA Kit (M5635-02; Omega Bio-Tek) according to the manufacturer’s protocol and stored at −20°C until further analysis. DNA concentration and purity were evaluated using a Qubit 4 Fluorometer (WiFi: Q33238) with Qubit 1X dsDNA HS Assay Kit (Q33231) and Qubit Assay Tubes (Q32856) (Invitrogen), while DNA integrity was assessed by agarose gel electrophoresis. Metagenomic shotgun libraries with an average insert size of 400 bp were prepared using the Illumina TruSeq Nano DNA LT Library Preparation Kit and sequenced on the Illumina NovaSeq platform (PE150; Illumina).

The quality control of each dataset was performed using FASTP to remove low quality sequences, splice contamination, etc [19]. The sequences after QC were aligned to the host genome using bowtie2 to get the aligned sam file. Then use samtools to filter out the sequences on the comparison, and get the double-ended sequences are the high-quality sequences that do not contain the information of the host gene [20,21]. Species annotation of reads was performed using Kraken2 [22], and abundance estimation was performed using Bracken [23], followed by species diversity analysis and differential species analysis based on abundance. Macrogenomic sequencing data assembly using MEGAHIT [24], assembly results can be summarized using QUAST [25]. Genes were identified and predicted from the assembled sequences using Prokka [26]. Gene sequence files obtained from the predictions of multiple samples were merged to construct a non-redundant gene set using CD-HIT [27]. Functional annotation of individual genes was conducted using eggNOG-mapper [28], while antibiotic resistance genes were identified based on the CARD database [29].

### Analysis of serum metabolome

Serum metabolome were analyzed using liquid chromatography (ACQUITY UPLCHSS T3) combined with mass spectrometer (Thermo Orbitrap Exploris 120). Data processing was performed with Compound Discoverer 3.3 (ver. 3.3.2.31; Thermo Fisher Scientific) and a custom-built PSNGM database, along with mzCloud (https://www.mzcloud.org/), LIPID MAPS (https://www.lipidmaps.org/), HMDB (https://hmdb.ca/), MoNA (https://mona.fiehnlab.ucdavis.edu/), and the NIST_2020_MSMS spectral library. These resources were used for raw peak extraction, baseline filtering, peak alignment, deconvolution analysis, peak identification, and area integration. Both mass spectrum and retention index matching were considered for metabolite identification. A total of 45 and 161 serum differential metabolites were identified in the early and late fattening stages, respectively. Metabolite datasets from the high and low groups in both stages were compared and visualized using the Pheatmap package (ver. 1.0.12) in R.

### Integrated analysis of microbiome and metabolome

Metagenomic sequencing of rumen fluid was performed, and differentially abundant metabolites were identified through untargeted serum metabolomics. Spearman correlation analysis was conducted to evaluate the associations between these metabolites and microbial genera. Correlations with |Spearman’s R|>0.50 and p<0.05 were considered statistically significant. Significantly correlated microbial genera and metabolites were subsequently visualized using a heatmap.

### Statistical analysis

Two-tailed Student’s t-tests were performed to compare variables between two groups, and Spearman’s correlation analysis was used to evaluate associations between two variables. A p-value<0.05 was considered statistically significant. Differentially abundant microbial species were identified using linear discriminant analysis effect size (LEfSe) [30], with significance defined by LDA>2 and p<0.05.

Principal component analysis (PCA) and orthogonal partial least squares discriminant analysis (OPLS-DA) were separately performed using the R package ropls to reduce the dimensionality of the sample data. p-value, OPLS-DA-derived variable importance in projection (VIP), and fold change (FC) were calculated to quantify the influence intensity and explanatory power of individual metabolite components on sample classification, facilitating the selection of discriminatory metabolites. Metabolites with p<0.05 and VIP>1 were considered statistically significant. Further receiver operating characteristic (ROC) curve analysis was performed using the pROC package (ver. 1.18.2) to extract key information from the differential metabolites. Functional analysis primarily involved Kyoto Encyclopedia of Genes and Genomes (KEGG) enrichment using the clusterProfiler package (ver. 4.6.0), which identified significantly enriched metabolic pathways and calculated overall differential abundance scores. These scores reflect the average and overall trend of changes in all differential metabolites within each pathway, thereby facilitating the identification of critical metabolic pathways.

## RESULTS

### Characterization of phenotypes

**Figure 2:**
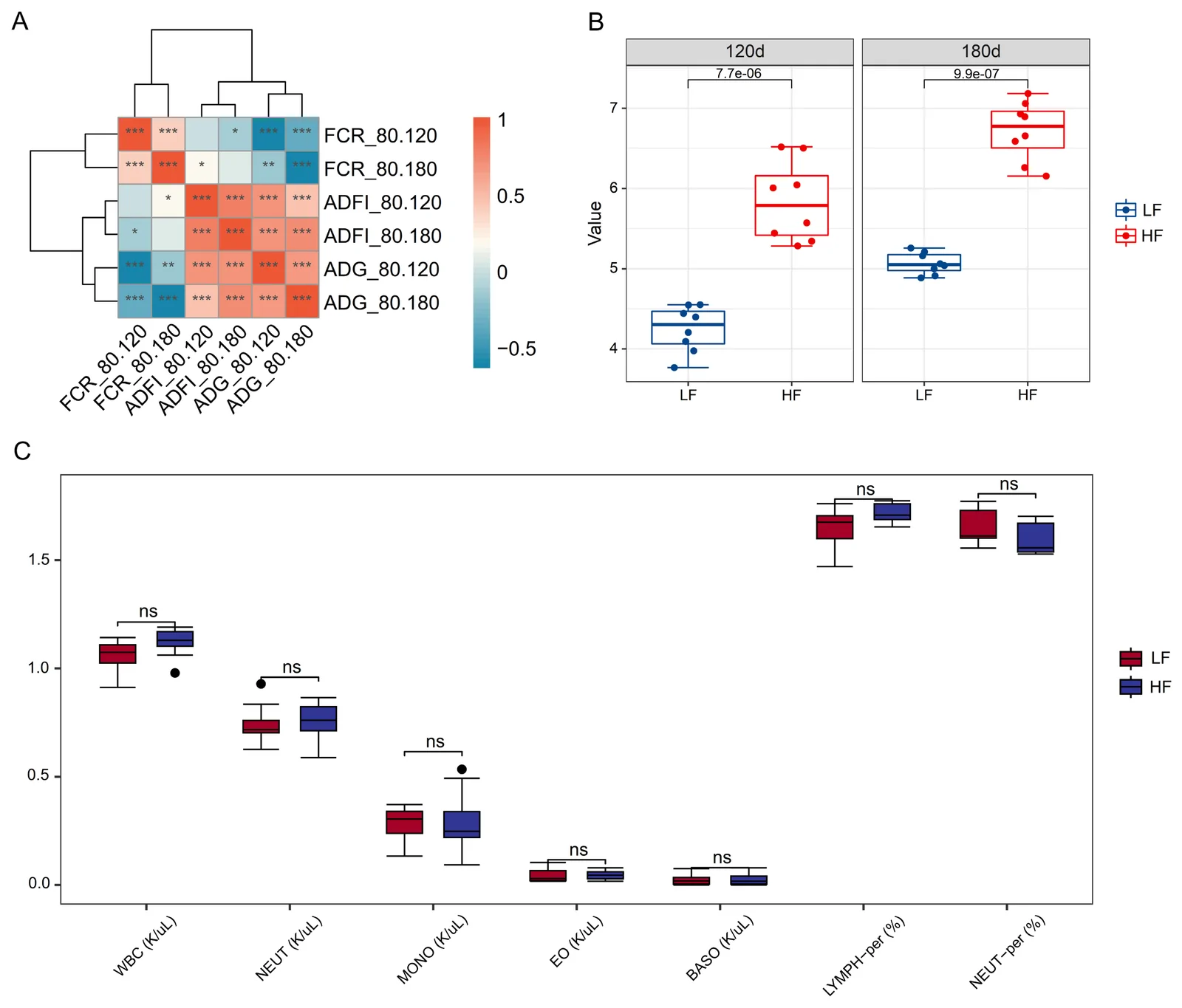
Correlation analysis and grouping of FCR. (A) Correlation between FCR and ADG, ADFI across two stages. (B) A highly significant difference in FCR was observed between the two stages (T1 and T2). (C) Differential analysis of blood physiological indexes between HF and LF at T2, “ns” indicates no significant difference. FCR, feed conversion ratio; ADFI, average daily feed intake; ADG, average daily gain; LF, lowest FCR group; HF, highest FCR group.

A correlation analysis of the phenotypes of all the sheep revealed FCR was significantly correlated with both average daily gain (ADG) and average daily feed intake (ADFI) across the two fattening stages (Figure 2A). In addition, FCR differed significantly between the HF and LF groups (p<0.05; Figure 2B), confirming the effectiveness of the grouping strategy. Blood physiological parameters measured in all sheep confirmed normal health status across groups, excluding disease as a confounding factor for FCR assessment (Figure 2C). These results provide a strong basis for the following microbiome and metabolome investigations.

### Profiling of the rumen fluid metagenome

Metagenome sequencing generated a total of 2,381,953,224 reads, with 74,436,038.25±1,621,150.275 reads (mean±standard error of the mean [SEM]) per sample, the average percentage of Q30 was 97%, and the average GC content was 45% (Supplement 2). After quality control and removing host genes, a total of 2,347,856,076 reads were retained, with 73,370,502.38 ±1,595,279.854 per sample, the average percentage of Q30 was 97.8%, and the average GC content was 44.8%. The rumen meta-genome consisted of 95.87% bacteria, 3.3% archaea, 0.814% viruses, and 0.011% eukaryote (Supplement 3A).

**Figure 3:**
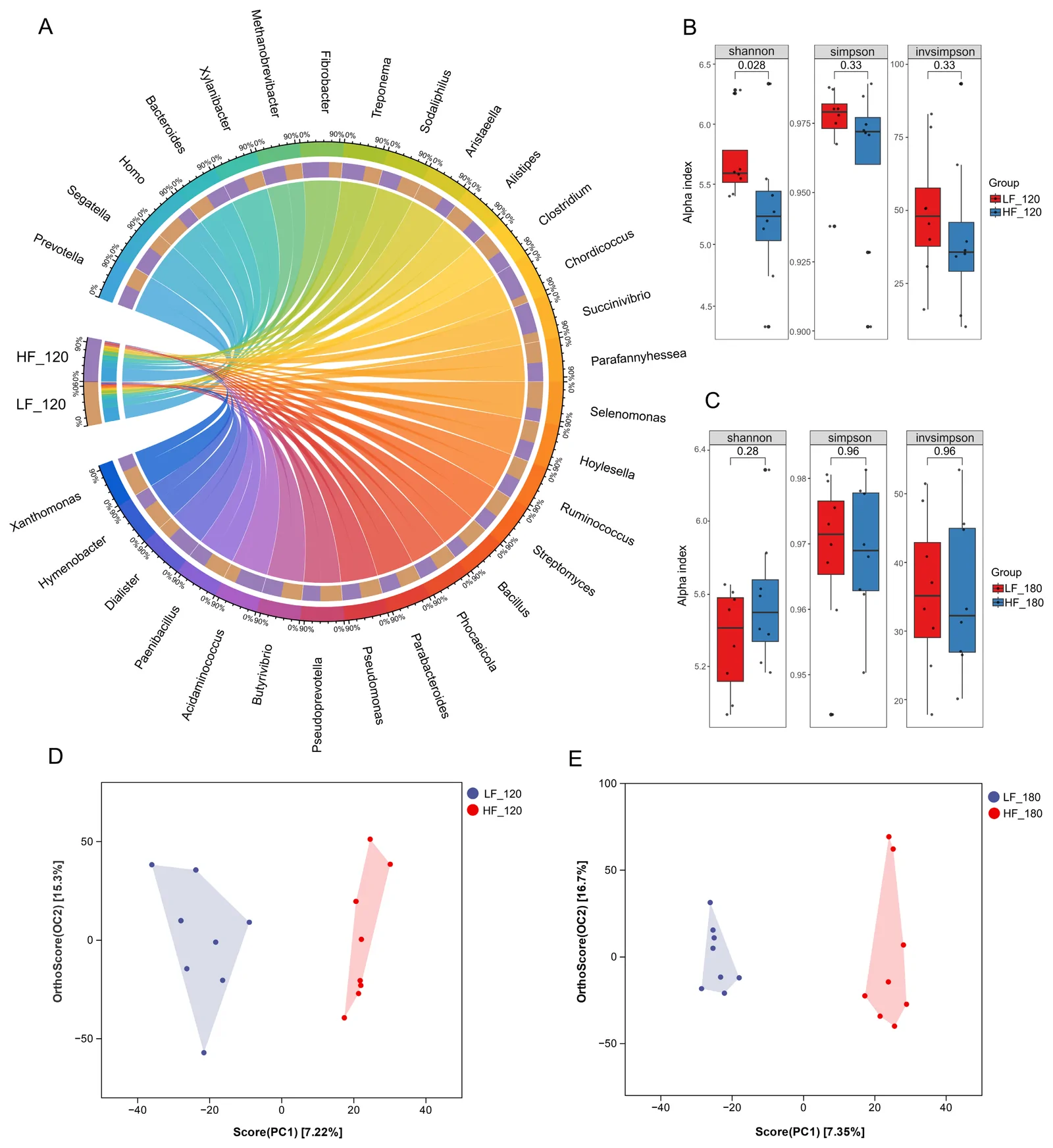
Characterization of the microbial composition and diversity between HF and LF groups. (A) Relative abundance of the top 30 genera at the genus level at T1. (B) Alpha diversity analysis of microbial communities at T1. (C) Alpha diversity analysis of microbial communities at T2. (D) OPLS-DA score plot of rumen microbiome between LF and HF groups at T1. PC1 represents Principal Component 1 and OC2 represents Principal Component 2, each point represents one sample, and different colored points indicate different grouping. (E) OPLS-DA score plot of rumen microbiome between LF and HF groups at T2. LF, lowest FCR group; HF, highest FCR group; FCR, feed conversion ratio; OPLS-DA, orthogonal partial least squares discriminant analysis.

Comparison of microbial composition changes between the HF and LF groups revealed that, at T1, genera such as *Bacteroides*, *Prevotella*, *Ruminococcus*, *Methylobacterium*, *Methanobrevibacter*, and *Methanosphaera* were identified (Figure 3A). At T2, *Bacteroides*, *Prevotella*, *Methanobrevibacter*, *Segatella*, and *Clostridium* were found (Supplement 3B). We performed an alpha diversity analysis for both stages and found that, at T1, there was a significant difference in the Shannon diversity index between the HF and LF groups, while no significant differences were observed for other parameters (Figure 3B). At T2, however, there were no significant differences in any of the diversity parameters (Figure 3C), suggesting a possible stabilization of the rumen microbial community over time, potentially minimizing individual FCR-related effects. PCA analyses were also performed for both stages, revealing no clear clustering between the HF and LF groups (Supplements 3D, 3E), but OPLS-DA analyses revealed significant clustering between the two groups (Figures 3D, 3E). In summary, metagenomic analysis of rumen fluid revealed dynamic shifts in microbial community composition and diversity between the HF and LF groups across different stages, suggesting a potential stabilization of the microbial ecosystem over time.

### Functional profiles of the rumen microbiome

In addition to analyzing highly abundant genera, we also examined other taxonomic levels. The dominant bacterial Phylum included *Bacteroidota*, *Bacillota*, and *Synergistota*, among others (Supplements 3E, 3F). To explore the microbial contribution to FCR, we performed species-level analysis.

**Figure 4:**
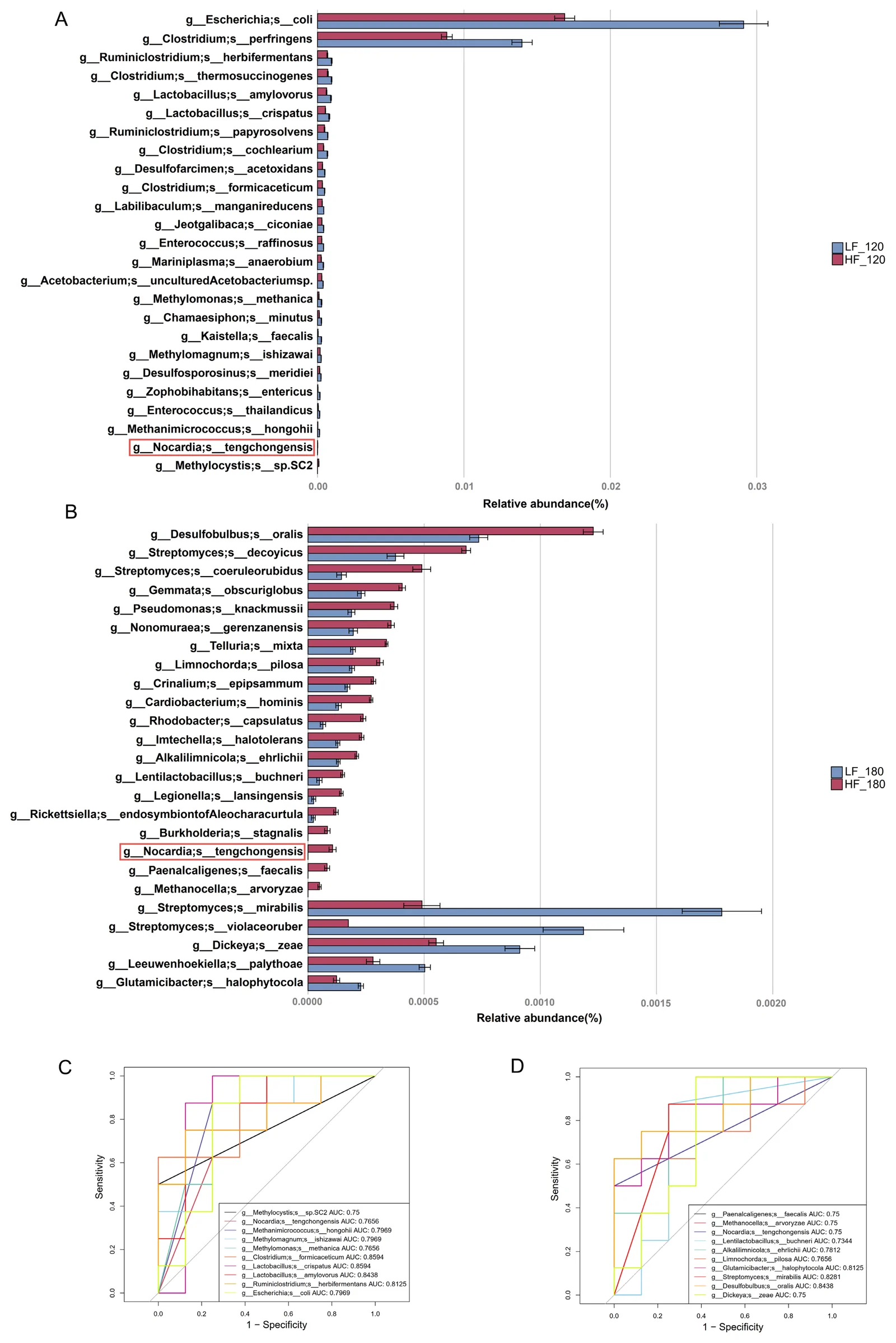
Differential rumen bacterial species between HF and LF groups. (A) Significantly different microbiota at T1. Significant differences were determined using LEfSe, with thresholds of LDA score>2 and p<0.05. Bar colors indicate group classification. (B) Significantly different microbiota at T2. (C) ROC curve analysis of key microbiota at T1. The x-axis represents the false positive rate (1–specificity), and the y-axis represents the true positive rate (sensitivity). A higher true positive rate and lower false positive rate indicate better model performance. The AUC quantifies the model’s predictive accuracy; values closer to 1.0 reflect stronger discriminatory power. (D) ROC curve analysis of key microbiota at T2. The red boxes indicate different microbiota shared between the two fattening stages (T1 and T2). LF, lowest FCR group; HF, highest FCR group; FCR, feed conversion ratio; LEfSe, linear discriminant analysis effect size; ROC, receiver operating characteristic; AUC, area under the curve.

At T1, 23 species exhibited significantly higher abundances in the LF group (LDA>2, p<0.05), including methane-related taxa such as *Methanimoccus hongohii*, *Methylomagnum ishizawai*, and *Methylomonas methanica*, as well as digestive metabolism-related species like *Ruminiclostridium herbifermentans* and *Escherichia coli* (Figure 4A). In contrast, only two species, namely *Nocardia tengchongensis* and *Methylocystis* sp. *SC2*, were enriched in the HF group at this stage. At T2, five species were enriched in the LF group, primarily actinomycetes such as *Streptomyces violaceoruber* and *Glutamicibacter halophytocola*, suggesting a strong association between LF-linked rumen microbiota and actinomycetes capable of producing growth-promoting secondary metabolites. Meanwhile, 20 species were significantly enriched in the HF group, including *Methanocella arvoryzae*, *Lentilactobacillus buchneri*, *Sporomusa ovata*, and *Desulfobulbus oralis* (Figure 4B). Notably, the *Nocardia tengchongensis* was found in both stages of the HF group. These results suggest that enrichment of fiber-degrading and metabolically efficient microbes in the LF group may enhance nutrient utilization and energy retention, whereas the higher abundance of methanogenic and lactate-associated taxa in the HF group may contribute to energy loss, thereby reducing feed efficiency. To evaluate the diagnostic potential of these species as biomarkers, ROC curve analysis was conducted. The area under the curve (AUC) values ranged from 0.734 to 0.859, indicating good discriminatory power between HF and LF groups (Figures 4C, 4D). This further supports the potential of these microbial taxa as biomarkers for the early prediction and nutritional management of sheep with poor feed efficiency.

### Functional annotated analysis of rumen microbiome between the HF and LF groups

**Figure 5:**
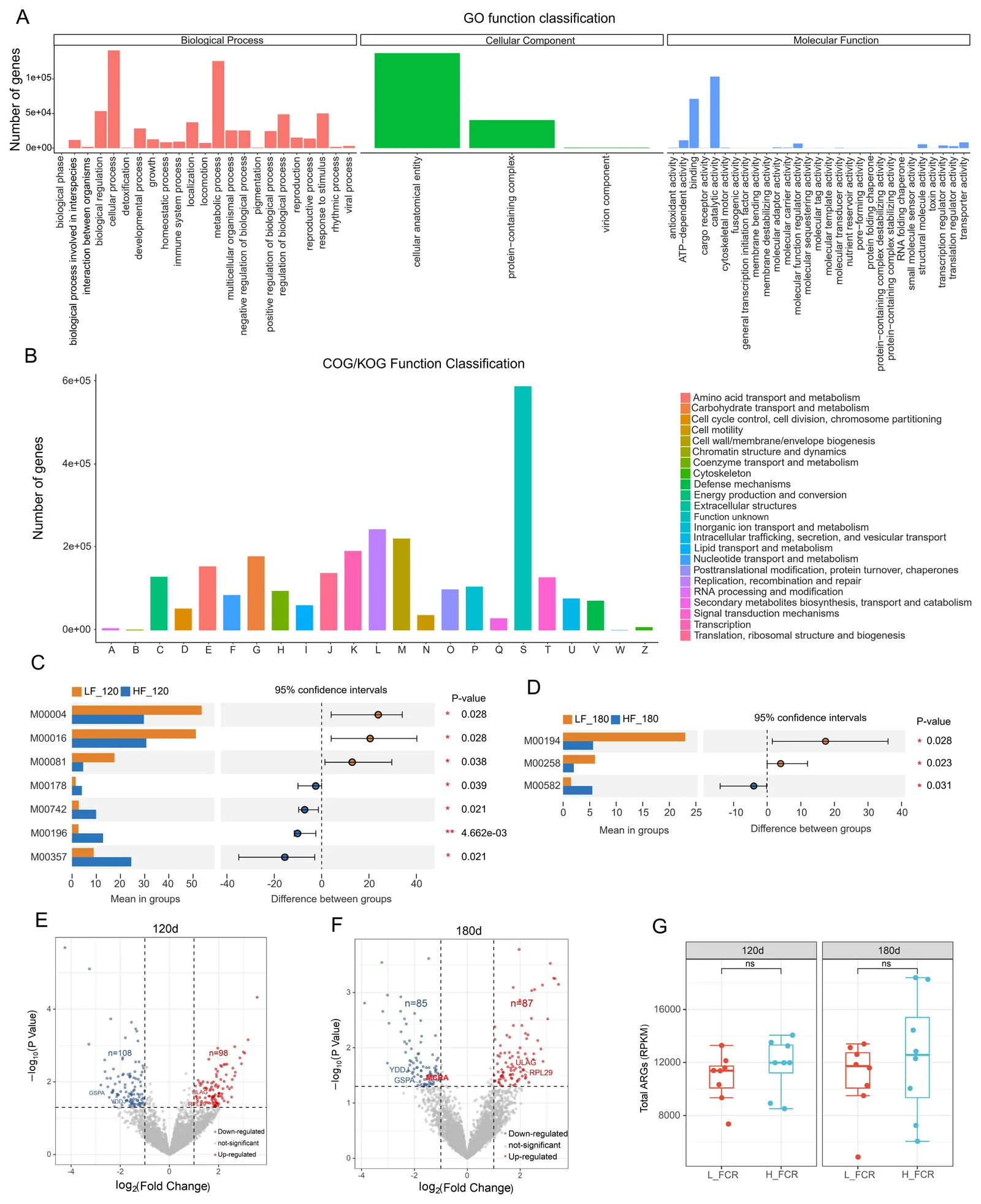
Functional annotation and differential expression analysis of rumen microbiota between HF and LF groups. (A) GO functional classification based on EggNOG annotation, revealing significant associations of FCR with metabolic and energy-related biological processes. (B) Functional classification based on COG and KOG. (C) Differential KEGG modules of rumen microbial between HF and LF cows at T1. (D) Differential KEGG modules of rumen microbial between HF and LF cows at T2. (E) Differential gene expression at T1. Blue dots indicate up-regulated genes, while red dots indicate down-regulated genes. (F) Differential gene expression at T2. Blue dots indicate up-regulated genes, while red dots indicate down-regulated genes. (G) CARD database functional annotation results. The vertical coordinate is the total abundance of ARGs in different groups. GO, Gene Ontology; LF, lowest FCR group; HF, highest FCR group; FCR, feed conversion ratio; KEGG, Kyoto Encyclopedia of Genes and Genomes.

Functional annotation was performed using the EggNOG database, incorporating Gene Ontology (GO) and Clusters of Orthologous Groups (COG/KOG) classifications. GO analysis revealed that FCR in sheep is closely linked to a range of biological processes, particularly those related to metabolism and energy (Figure 5A). COG/KOG analysis further indicated significant differences in functional categories between the HF and LF groups, notably enriched categories included amino acid transport and metabolism (Figure 5B). These findings underscore the complex biological roles of the rumen microbiota in shaping feed efficiency.

After performing functional annotation using EggNOG to obtain specific gene names. Based on the annotation outcomes, a differential expression analysis was conducted. When comparing groups with distinct FCR, five functional pathways were identified as significantly more active in LF group than in HF group (p<0.05). These included the Pentose phosphate pathway (M00004), Lysine biosynthesis via the succinyl-DAP pathway (M00016), and Pectin degradation (M00081). Conversely, five pathways were significantly more active in the HF group (p<0.05), notably Methanogenesis from acetate to methane (M00357) (Figures 5C, 5D). These findings suggest that enhanced carbohydrate metabolism and biosynthetic pathways in the LF group may improve energy utilization efficiency, whereas increased methanogenesis in the HF group may lead to energy loss in the form of methane, thereby contributing to poorer feed efficiency.

We initially filtered genes from the Prokka annotation results based on high-confidence criteria: an E-value threshold of less than 1×10^−5^ and a score greater than 100. Following this, differential expression analysis was performed using gene names from the ‘Preferred_name’ column. Differential gene expression at T1 identified 108 up-regulated and 98 down-regulated genes in the LF group relative to the HF group (Figure 5E). At T2, 85 genes were up-regulated and 87 down-regulated in the LF group (Figure 5F). Genes consistently up-regulated in the LF group included *GSPA* and *YDDJ*, whereas *ULAG* and *RPL29* were down-regulated. Importantly, *MCRA*, a gene implicated in methanogenesis, was found to be highly expressed in the HF group.

To evaluate whether feed efficiency correlates with antibiotic-resistance potential, we profiled ARGs with the CARD database. No significant associations were detected (Figure 5G), suggesting that antibiotic resistance is unlikely to be a major factor influencing feed efficiency under the controlled experimental conditions.

### Serum metabolite profiling and differential analysis between the HF and LF groups

**Figure 6:**
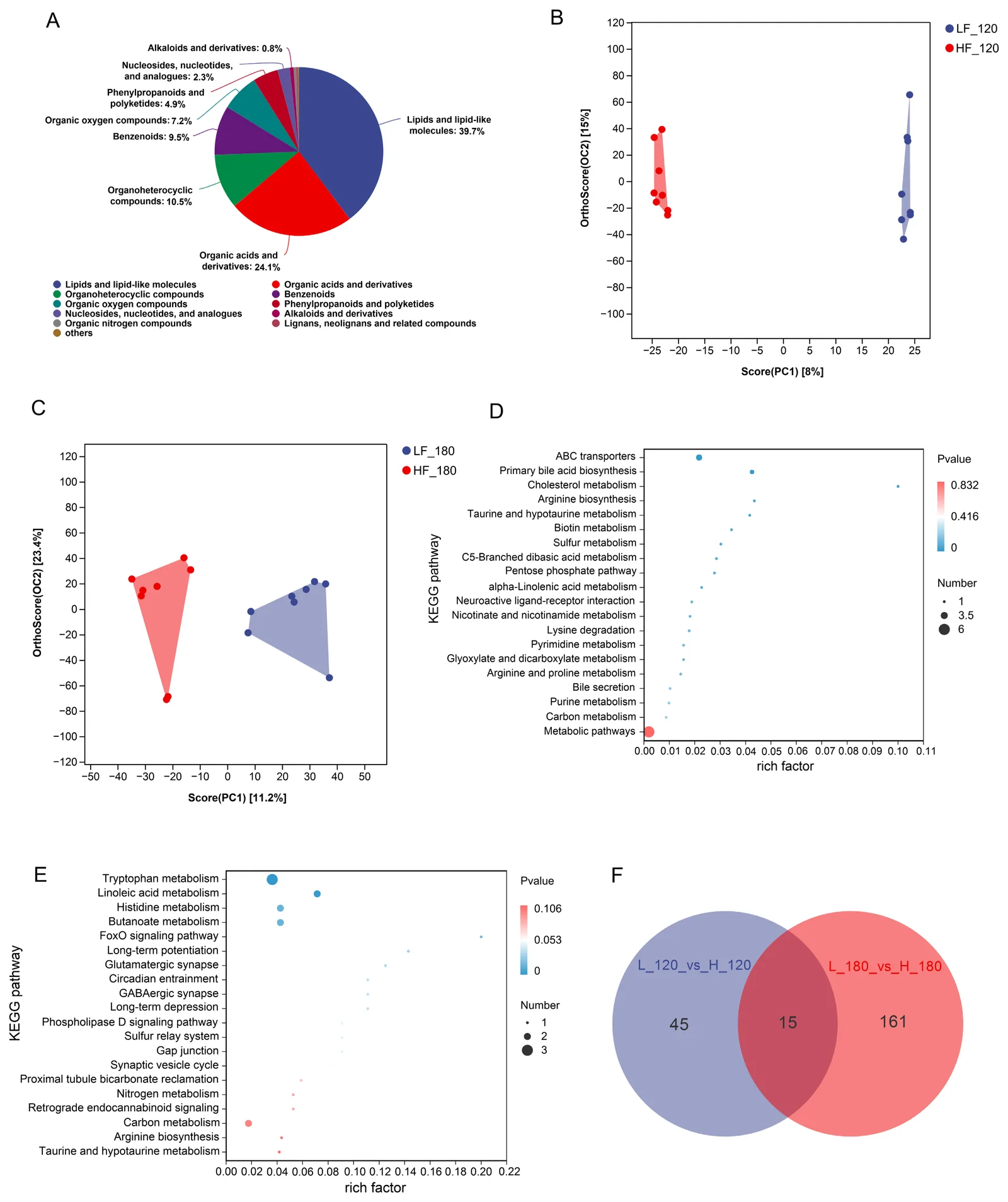
Serum metabolite profiling and differential analysis between the HF and LF group. (A) The different color blocks in the figure express different chemical classification attribution entries, and the percentage represents the number of metabolites in that chemical classification attribution entry as a percentage of the number of all identified metabolites. The figure mainly shows the top 10 classifications. (B) OPLS-DA score plot of serum metabolome between LF and HF groups at T1. PC1 represents Principal Component 1 and OC2 represents Principal Component 2, each point represents one sample, and different colored points indicate different grouping. (C) OPLS-DA score plot of serum metabolome between LF and HF groups at T2. (D) Enrichment analysis of differential metabolites at T1. The top 20 KEGG pathways with the lowest FDR values are shown. Enrichment was evaluated by Rich factor, FDR, and the number of metabolites mapped to each pathway. A higher Rich factor indicates greater enrichment; lower FDR values indicate higher significance. (E) Enrichment analysis of differential metabolites at T2. (F) Overlapping differential metabolites between the two stages. LF, lowest FCR group; HF, highest FCR group; FCR, feed conversion ratio; KEGG, Kyoto Encyclopedia of Genes and Genomes; OPLS-DA, orthogonal partial least squares discriminant analysis.

A total of 10 major classes of compounds were identified in the serum metabolome (Figure 6A). While PCA analyses for both stages revealed no clear clustering between the HF and LF groups (Supplements 4A, 4B), OPLS-DA analysis showed a clear separation between the two groups at both stages (Figures 6B, 6C). This indicates strong differentiation between the HF and LF groups. At T1, 41 and 19 differential metabolites were identified at positive-ion and negative-ion modes, respectively. At T2, 105 and 71 differential metabolites were identified at positive-ion and negative-ion modes, respectively. We enriched and analyzed the differential metabolites in the two stages separately. At T1, the identified pathways included substance transport, primary bile acid and cholesterol biosynthesis and metabolism, amino acid synthesis and degradation, other metabolic processes, and signaling pathways (Figure 6D). These pathways are closely related to lipid digestion, nutrient transport, and amino acid metabolism, which may influence energy availability and growth performance, thereby affecting FCR. At T2, the pathways involved included tryptophan, linoleic acid, histidine, and butyric acid metabolism, as well as the FoxO signaling pathway (Figure 6E). In particular, pathways such as butyrate and linoleic acid metabolism are involved in energy supply, while amino acid metabolism and FoxO signaling are associated with growth regulation and metabolic efficiency, suggesting their potential roles in modulating feed efficiency. Notably, 15 differential metabolites were identified as significant in both groups (Figure 6F). These shared metabolites may represent core metabolic factors contributing to feed efficiency across different fattening stages. Correlation analysis of differential metabolites between the two stages helps to further elucidate the inter-regulatory relationships among metabolites during biological state changes. Metabolites that show correlated expression may participate in the same biological processes, providing insights into their functional roles (Supplements 4C, 4D). Such coordinated metabolic changes may reflect integrated regulation of energy utilization and growth processes associated with FCR variation.

**Figure 7:**
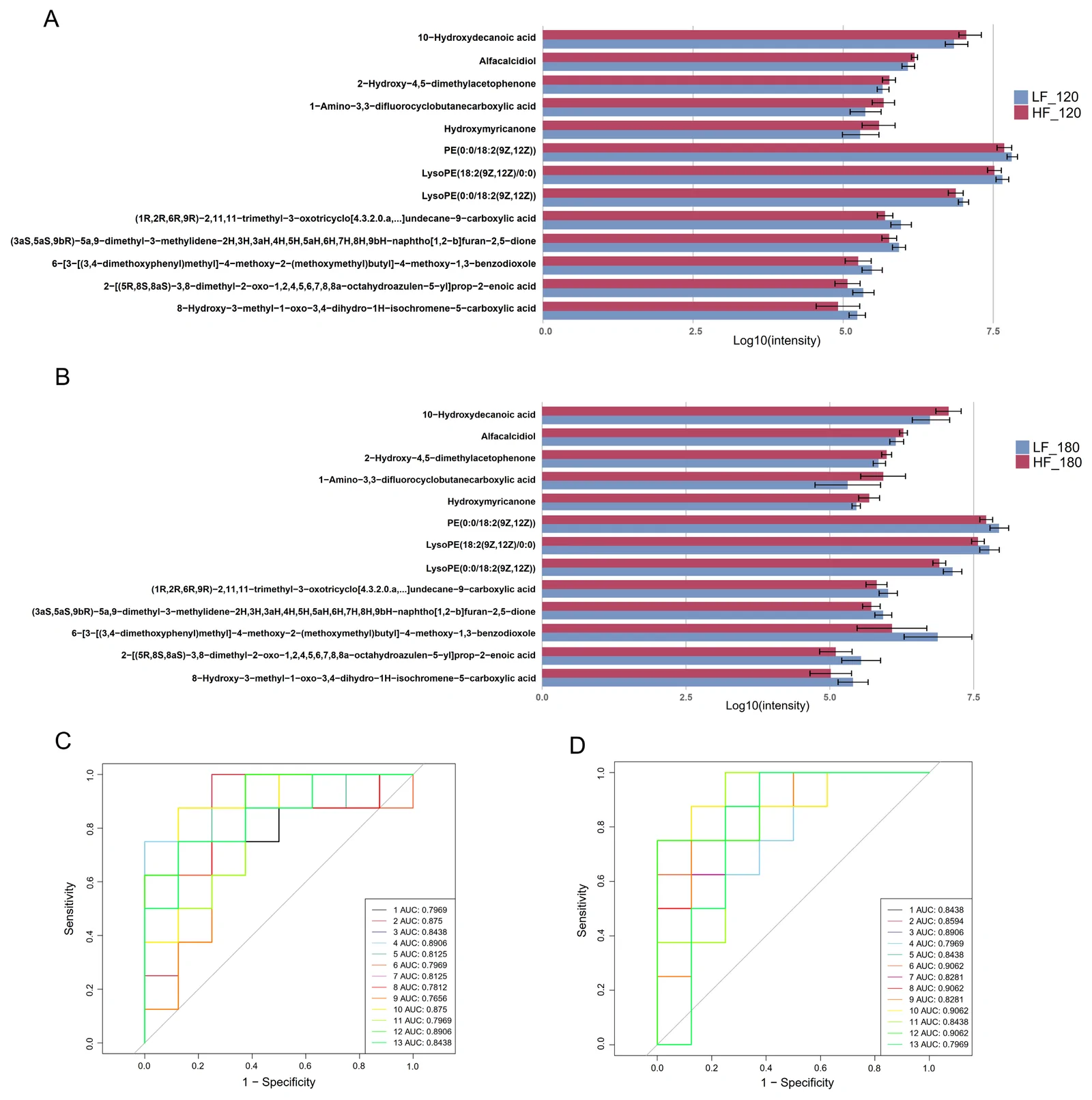
Differential serum metabolites between HF and LF groups. (A) The x-axis shows log-transformed metabolite signal intensities, and the y-axis displays metabolite names. Bar colors indicate group classification. Metabolites with p<0.05 were considered significantly different. (B) Significantly different metabolites at T2. (C) ROC curve analysis of different metabolites at T1. The x-axis represents the false positive rate (1–specificity), and the y-axis represents the true positive rate (sensitivity). A higher true positive rate and lower false positive rate indicate better model performance. The AUC quantifies the model’s predictive accuracy; values closer to 1.0 reflect stronger discriminatory power. Numbers 1–13 in the figure correspond to the metabolites listed from top to bottom in Figure 6A. (D) ROC curve analysis of different metabolites at T2. Numbers 1–13 in the figure correspond to the metabolites listed from top to bottom in Figure 6B. LF, lowest FCR group; HF, highest FCR group; FCR, feed conversion ratio; AUC, area under the curve; ROC, receiver operating characteristic.

The 13 common differential metabolites showed consistent trends in both stages, with eight differential metabolites in the LF group having significantly higher abundance than the HF group, and five differential metabolites having significantly lower abundance than the HF group (Figures 7A, 7B). Following this, ROC curve analysis was conducted separately for the differential metabolites in both stages. The results revealed that the AUC values for each differential metabolite in both stages were above 0.76, which had better accuracy (Figures 7C, 7D). These serum metabolites may serve as robust biomarkers for feed conversion efficiency and could potentially assist in the early prediction of feed efficiency in sheep.

### Comparative analysis between LF groups

**Figure 8:**
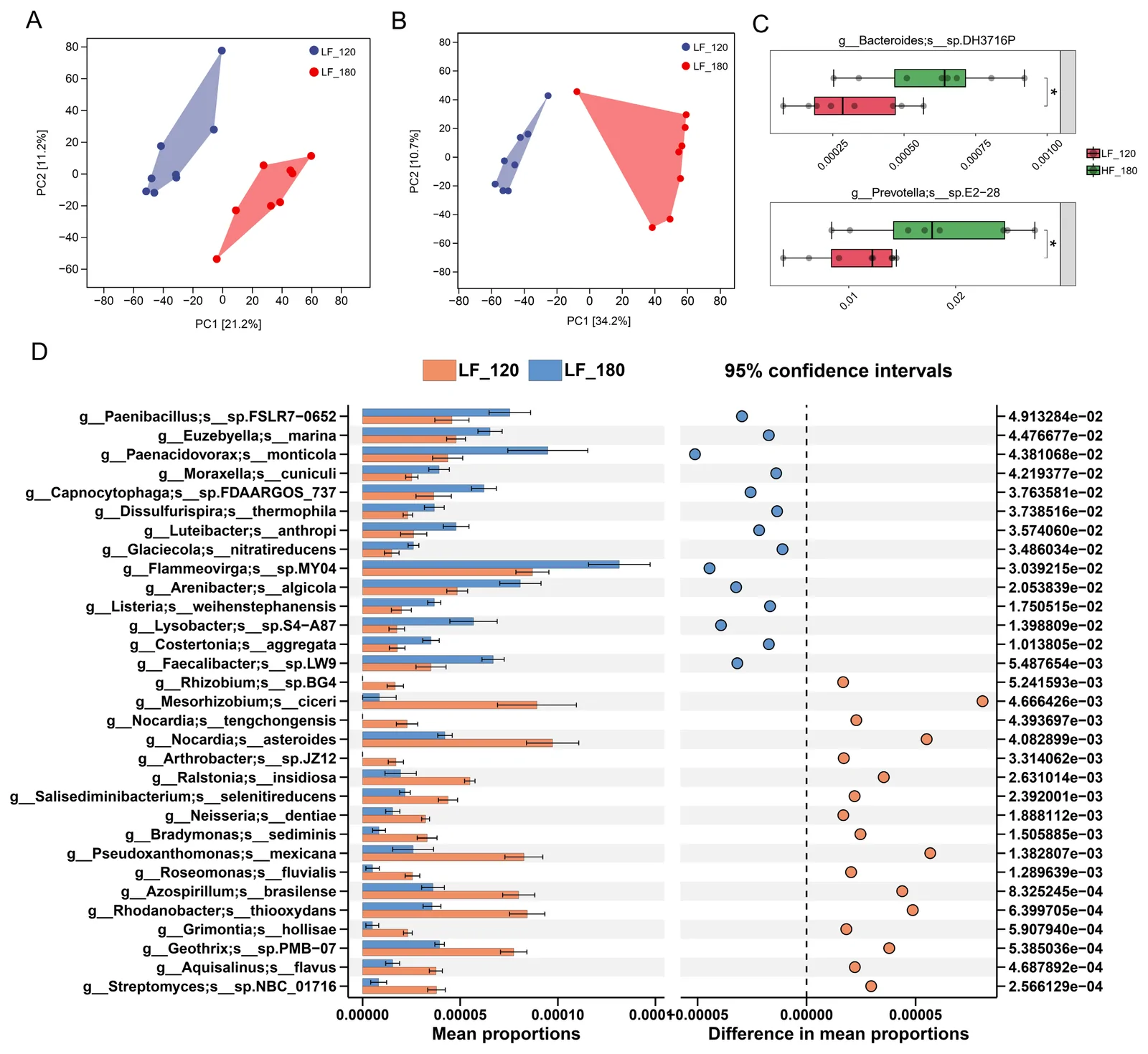
Comparative analysis of rumen microbiota and metabolites between LF groups. (A) PCA score plot of rumen microbiome between LF groups. PC1 represents Principal Component 1 and PC2 represents Principal Component 2, each point represents one sample, and different colored points indicate different groupings. (B) PCA score plot of serum metabolome between LF groups. (C) Differential abundance of key microbial species between T1 and T2. (D) Comparison of differential microbial species within the LF groups. Significantly different taxa between T1 and T2 were identified using the Wilcoxon rank-sum test (p<0.01). LF, lowest FCR group; HF, highest FCR group; FCR, feed conversion ratio; PCA, principal component analysis.

To further explore the FCR, further comparisons were made between LF groups in the early fattening stage and late fattening stage. The rumen microbes and metabolome were better classified with significant clustering compared to the previous PCA (Figures 8A, 8B). Initially, we concentrated on *Bacteroides* and *Prevotella* due to their significant role in gut health. Our results indicated that *Bacteroides* sp. *DH3716P* and *Prevotella* sp. *E2–28* were markedly less abundant in the early fattening stage than in the late fattening stage (Figure 8C). This implies that the populations of *Bacteroides* sp. *DH3716P* and *Prevotella* sp. *E2–28* rise with growth and development, showing a positive correlation. Moreover, *Faecalibacterium* sp. *LW9* and *Luteibacter anthropi* were found to be up-regulated with growth. Concomitantly, we observed a downregulation in the abundance of several microbial species during the early fattening stage as growth progressed, include *Streptomyces* sp. *NBC_01716*, *Grimontia hollisae*, *Rhodanobacter thiooxydans*, *Azospirillum brasilense*, and *Rhizobium* sp. *BG4* (Figure 8D). These species are primarily involved in nitrogen fixation, growth regulation, metabolism, and maintaining intestinal health.

A total of 167 (positive mode) and 144 (negative mode) differential serum metabolites were identified, collectively enriched in metabolic and fatty acid synthesis/degradation pathways (Supplements 5A, 5B). These pathways are closely associated with energy production and lipid utilization, suggesting their potential roles in modulating feed efficiency through differences in energy metabolism.

### Correlation analysis of microbiome and metabolome, and their explainabilities for feed conversion ratio

**Figure 9:**
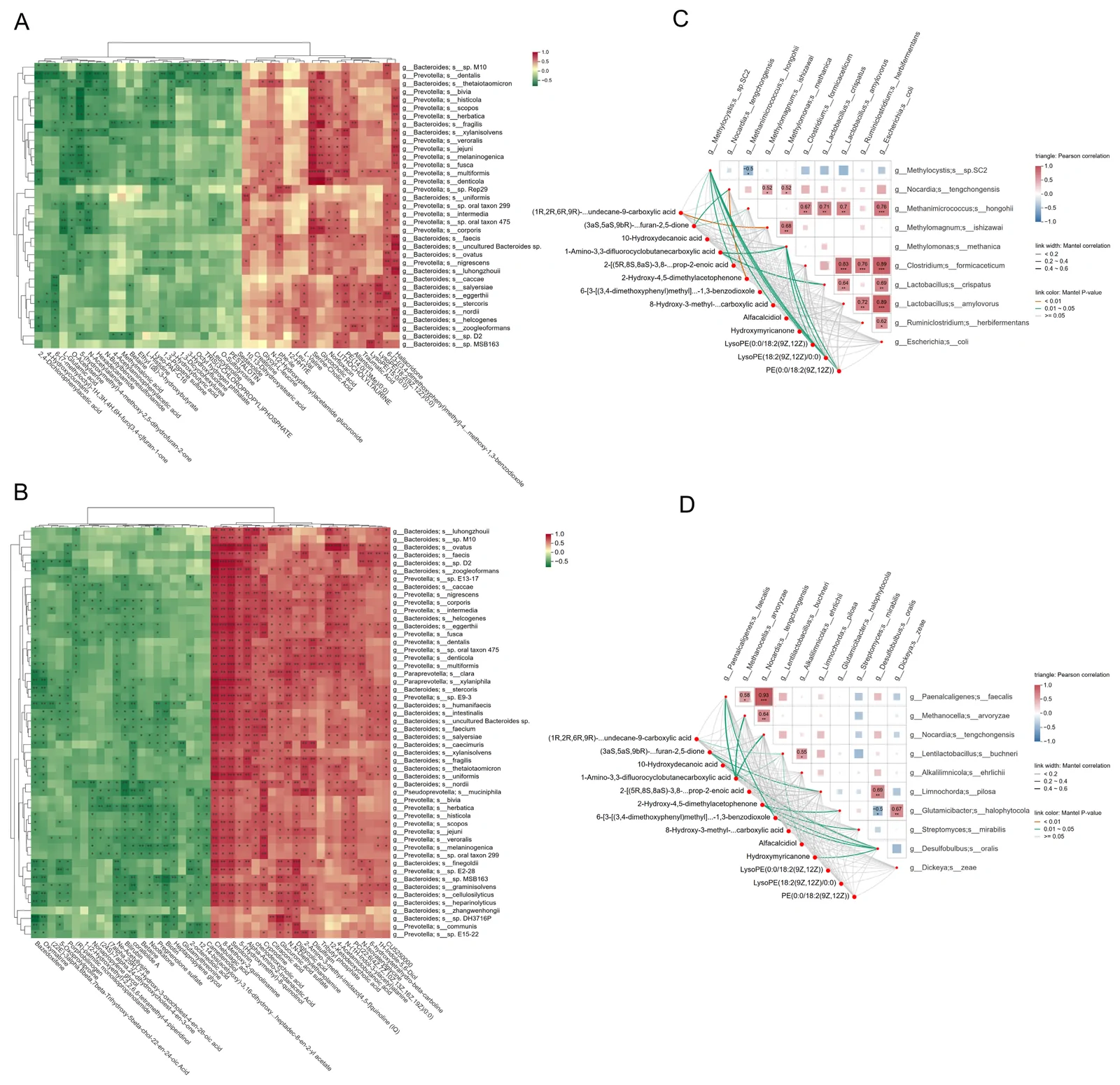
Correlation analysis of microorganisms and metabolites between HF and LF groups. (A) Spearman’s correlations between rumen microorganisms and serum metabolites at T1. (B) Spearman’s correlations between rumen microbiota and serum metabolites at T2. (C) Spearman’s correlations between correlation analysis of differential microorganisms and differential metabolites at T1. (D) Spearman’s correlations between correlation analysis of differential microorganisms and differential metabolites at T2. HF, highest FCR group; LF, lowest FCR group; FCR, feed conversion ratio.

At T1, Spearman’s rank correlations between the rumen microbiota and rumen metabolites were assessed, with the results revealing 81 significant correlations (R>0.50, p<0.05; Figure 9A). These correlations involved 18 *Prevotella*, 18 *Lactobacillus*, and 1 *Methanobacterium* species, and 44 metabolites. The results showed that these microorganisms were negatively correlated with 25 metabolites, mainly related to the metabolism of ureas, amino acids, peptides, and analogues, glycerophosphocholines, and fatty amides compounds. On the contrary, there was a positive correlation between these microorganisms and 19 other metabolites, mainly involving the metabolism of fatty amides, glycerophosphoethanolamines, carbohydrates and carbohydrate conjugates (Supplement 6). These associations suggest that key rumen microbes such as *Prevotella* and *Lactobacillus* may influence feed efficiency by modulating carbohydrate and nitrogen metabolism, thereby affecting energy extraction and nutrient utilization in the host. Similarly, At T2, Spearman’s rank correlations between the rumen microbiota and rumen metabolites were assessed, with the results revealing 96 significant correlations (Figure 9B). These correlations involved 22 *Prevotella* species, 28 *Bacteroides* species, 2 *Paraprevotella* species, and 44 metabolites. Results indicated that these microbes and metabolites were negatively correlated, primarily involving the metabolism of steroids, terpenoids, bilirubin, and biologically active compounds. Conversely, positive correlations were found between these microbes and 22 other metabolites, mainly associated with the metabolism of acids, alcohols, carbohydrates, phosphate esters, and tryptophan (Supplement 7). Such interactions indicate that shifts in microbial composition may alter the metabolism of lipids, amino acids, and bioactive compounds, potentially impacting energy balance and metabolic efficiency during later fattening stages. However, we found an increase in the number of correlated flora, suggesting that the increased complexity of microbe–metabolite interactions may reflect a more active metabolic network, which could contribute to differences in feed efficiency at later stages.

Correlation analysis was done to further investigate the relationship between biomarkers of rumen microbes and biomarkers of serum metabolites. At T1, the results indicated significant correlations between certain microbiota and common metabolites. Significant correlations between *Methylocystis* sp. *SC2* and several metabolites, including Hydroxymyricanone and the linoleic acid–containing LysoPE species LysoPE (0:0/18:2(9Z,12Z)) and LysoPE(18:2(9Z,12Z)/0:0), indicate that this taxon may act as a metabolic regulatory node (Figure 9C). At T2, Notably, several taxa, including *Paenalcaligenes faecalis*, *Methanocella arvoryzae*, and *Nocardia tengchongensis*, were significantly associated with the single metabolite 1-Amino-3,3-difluorocyclobutanecarboxylic acid (Figure 9D), suggesting it functions as a common metabolic node among these species. Together, these findings suggest that interactions between rumen microbiota and metabolites may influence feed efficiency by regulating nutrient metabolism, energy utilization, and host metabolic responses in a stage-specific manner.

## DISCUSSION

FCR remains the primary target for improving ruminant production efficiency. By simultaneously profiling rumen microbiota and host serum metabolites across two fattening stages, we provide a multi-omics view of the biological processes that govern feed efficiency in sheep.

Similar to many previous studies that have investigated the energy harvest efficiency of ruminants using metagenomic, bacteria were the most abundant rumen microbial kingdom in the rumen of sheep and the differences in the rumen microbial features between LF and HF sheep were mainly found in bacteria. Interestingly, alpha diversity analysis showed a significant difference in microbial diversity between HF and LF groups at the early fattening stage but not at the late fattening stage. This suggests that microbial community stabilization may reduce inter-individual variability over time [31]. Even under identical feeding conditions, individual differences in rumen fermentation and host metabolic status may create distinct microbial niches during the early fattening stage. However, despite the reduced microbial differences at the late fattening stage, significant differences in FCR persisted, indicating that host-related factors, including genetic background and metabolic regulation, may also contribute to feed efficiency variation. These findings aligned with prior studies that indicated microbial differences were more pronounced early in life and that microbes became progressively more abundant as organisms grew and developed [32–34].

Functionally, LF group were enriched in modules related to energy generation, amino acid biosynthesis and secondary metabolite production, while HF group showed higher activity of ABC transporter and protease-regulatory pathways, reflecting greater energy expenditure for nutrient acquisition. Elevated expression of the methanogenesis marker *MCRA* in HF sheep further supports that methane production is coupled to poor feed efficiency. Therefore, nutritional strategies aimed at reducing methanogenesis-related energy loss and promoting metabolically efficient rumen microbes may help improve feed utilization in HF sheep. This result is consistent with prior research indicating that ruminants with lower methane emissions possess distinct microbial metabolic profiles, leading to enhanced feed efficiency [35–37]. Notably, 13 differential metabolites were consistently identified across both feeding stages, with AUC>0.76, underscoring their potential as predictive biomarkers.

In correlation analyses, *Bacteroides* and *Prevotella* were found to show significant correlations with a number of metabolites, suggesting a closer relationship between these two genera and FCR. Many studies have shown that FCR is closely related to *Bacteroides*, *Prevotella* [38,39].

Although our study largely controlled for factors affecting FCR in sheep, including feed, management, and age. We found that FCR is related to changes in rumen microbes and their metabolites, as well as host utilisation and absorption of metabolites. In addition to these factors, FCR may be genetically related to the genetic mechanism of feed efficiency through genomic level studies, which needs to be further confirmed. In addition, recent amplicon sequencing-based studies have reported that genetics in ruminants not only influences phenotype but also rumen microbiota, and that heritable microbial taxa are associated with feed efficiency and methane emissions [13,40]. Due to the lack of understanding of the heritability of microbial functions and host metabolites, future studies need to assess the heritability of these functional and metabolic elements to further optimise feed utilisation efficiency and reduce greenhouse gas emissions in ruminants.

## CONCLUSION

This study integrated rumen microbiome profiling and host metabolomics to investigate the biological drivers of FCR in Hu sheep.

Our results demonstrated that differences in FCR are closely associated with distinct rumen microbial compositions and metabolic profiles. The LF group was characterized by enrichment of beneficial microbial taxa involved in fiber degradation and energy metabolism, whereas the HF group showed greater abundance of methane-associated and less efficient metabolic pathways, indicating increased energy loss. Metabolomic analysis further revealed that FCR is closely linked to lipid metabolism, amino acid metabolism, and bile acid biosynthesis. Correlation analysis confirmed strong interactions between key rumen microbes (e.g., Prevotella, Lactobacillus, Methanobacterium) and host metabolites, suggesting coordinated microbial–metabolic regulation of feed efficiency. Overall, these findings provide new insights into the microbial and metabolic mechanisms underlying feed efficiency and offer potential targets for improving livestock production performance through rumen microbiome modulation.

## SUPPLEMENTARY MATERIAL

**Supplement 1.** Diet and animal cohort information.

**Supplement 2.** QC results of metagenome sequencing data.

**Supplement 3.** Overview of rumen metagenomics composition and diversity.

**Supplement 4.** Differential metabolites correlation analysis.

**Supplement 5.** Functional enrichment of differential metabolites.

**Supplement 6.** Metabolic pathways of some metabolites at T1.

**Supplement 7.** Metabolic pathways of some metabolites at T2.
