# Supplementary material for: Integrating rumen microbiome and host metabolome to investigate feed conversion ratio across different fattening stages in Hu sheep
Source: Anim Biosci. 2026 Jun 1;39(9):260317. doi: 10.5713/ab.260317 (PMC13547752; doi:10.5713/ab.260317)
Supplement: Supplementary file 1 [file ab-260317-Supplementary-1.pdf]

Supplement 1. Diet and animal cohort information

Ingredient composition (% as fed)		Chemical composition	
Corn	32.5	Dry matter (DM) [%]	88.78
Corn germ meal	18	Crude protein (CP) [%]	13.09
Corn stalks	12	Digestible energy [MJ/kg]	11.11
Corn hulls	11.2	Neutral detergent fiber (NDF) [%]	27.08
Corn cob	8	Acid detergent fiber (ADF) [%]	13.99
Soybean meal	5	Crude fiber (CF) [%]	9.78
Cotton meal	5		
Molasses	3.3		
Bentonite	1.5		
Baking soda	1		
Stone powder	0.8		
Expanded Urea	0.5		
Premix	0.5		
