# Supplementary material for: Integrating rumen microbiome and host metabolome to investigate feed conversion ratio across different fattening stages in Hu sheep
Source: Anim Biosci. 2026 Jun 1;39(9):260317. doi: 10.5713/ab.260317 (PMC13547752; doi:10.5713/ab.260317)
Supplement: Supplementary file 2 [file ab-260317-Supplementary-3.pdf]

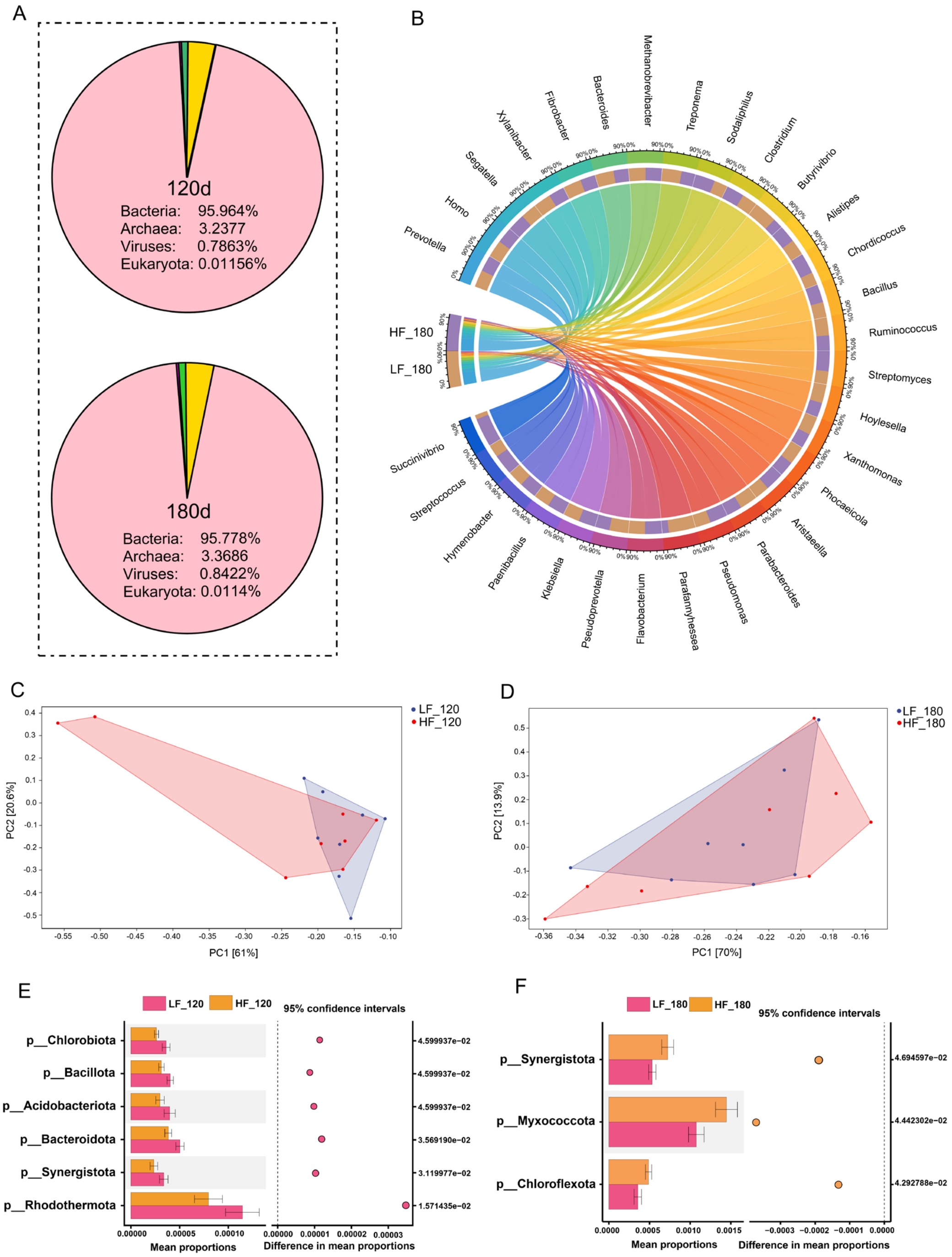

Supplement 3. Overview of rumen metagenomics composition and diversity. (A) Taxonomic composition of the rumen metagenome at T1 and T2. (B) Relative abundance of the top 30 genera at the genus level at T2. (C) PCA score plot of rumen microbiome between HF and LF groups at T1. PC1 represents Principal Component 1 and PC2 represents Principal Component 2, each point represents one sample, and different colored points indicate different groupings. (D) PCA score plot of rumen

microbiome between HF and LF groups at T2. (E) Differential microbial phyla between HF and LF groups at T1. Significant differences were identified using the Wilcoxon rank-sum test ($P < 0.01$). (F) Differential microbial phyla between HF and LF groups at T2. Significant differences were identified using the Wilcoxon rank-sum test ($P < 0.01$).
