# Supplementary material for: Integrating rumen microbiome and host metabolome to investigate feed conversion ratio across different fattening stages in Hu sheep
Source: Anim Biosci. 2026 Jun 1;39(9):260317. doi: 10.5713/ab.260317 (PMC13547752; doi:10.5713/ab.260317)
Supplement: Supplementary file 3 [file ab-260317-Supplementary-4.pdf]

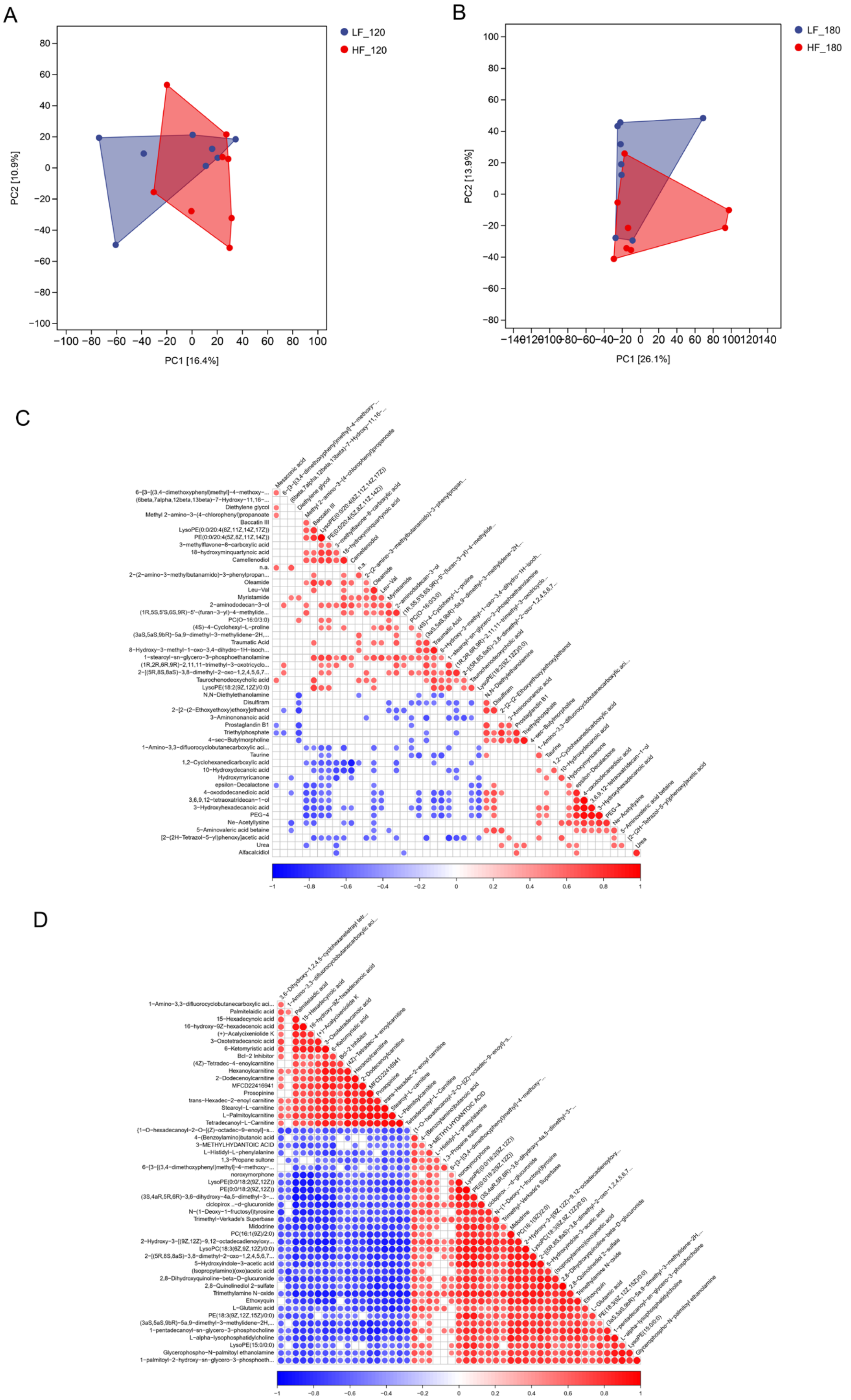

Supplement 4. Differential metabolites correlation analysis. (A) PCA score plot of serum metabolome between HF and LF groups at T1. PC1 represents Principal Component 1 and PC2 represents Principal Component 2, each point represents one sample, and different colored points indicate different groupings. (B) PCA score plot of serum metabolome between HF and LF groups at T2. (C) Correlation analysis of differential

metabolites at T1. Both the vertical and diagonal axes represent differential metabolite names. The color gradient indicates correlation strength, with red denoting positive correlation and blue indicating negative correlation. Darker shades correspond to stronger correlations. (D) Correlation analysis of differential metabolites at T2.
