# Supplementary material for: Integrating rumen microbiome and host metabolome to investigate feed conversion ratio across different fattening stages in Hu sheep
Source: Anim Biosci. 2026 Jun 1;39(9):260317. doi: 10.5713/ab.260317 (PMC13547752; doi:10.5713/ab.260317)
Supplement: Supplementary file 4 [file ab-260317-Supplementary-5.pdf]

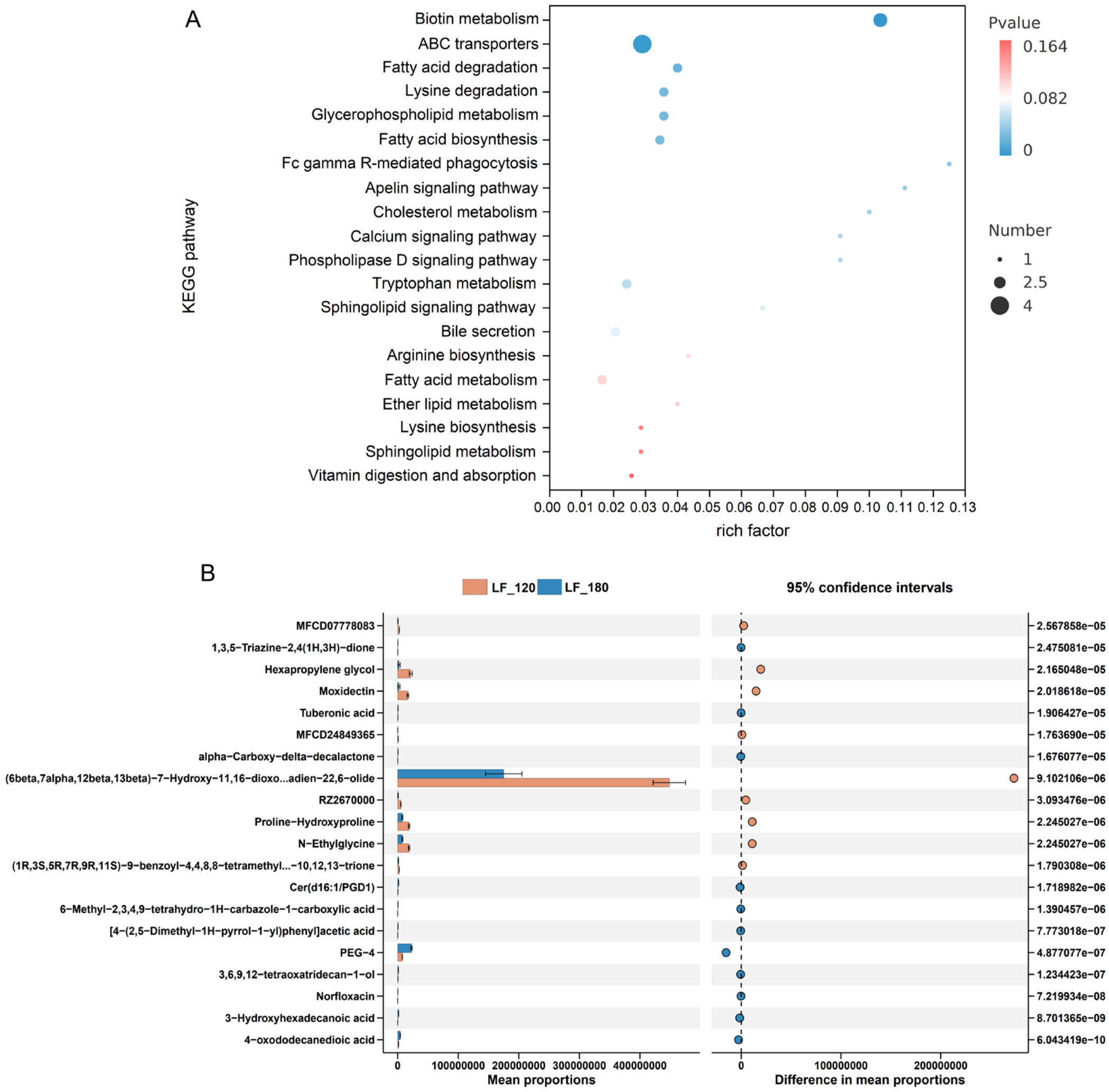

Supplement 5. Functional enrichment of differential metabolites. (A) KEGG pathway enrichment analysis of differential metabolites. The top 20 KEGG pathways with the lowest FDR values are shown. Enrichment was evaluated by Rich factor, FDR, and the number of metabolites mapped to each pathway. A higher Rich factor indicates greater enrichment; lower FDR values indicate higher significance. (B) Comparison of differential metabolites within the LF groups. Significantly different metabolites between T1 and T2 were identified using the Wilcoxon rank-sum test ($P < 0.01$).
